# Converting quadratic entropy to diversity: Both animals and alleles are diverse, but some are more diverse than others

**DOI:** 10.1371/journal.pone.0185499

**Published:** 2017-10-31

**Authors:** Peter E. Smouse, Sam C. Banks, Rod Peakall

**Affiliations:** 1 Department of Ecology, Evolution & Natural Resources, Rutgers University, New Brunswick, New Jersey, United States of America; 2 The Fenner School of Environment and Society, The Australian National University, Acton, ACT, Australia; 3 Research School of Biology, The Australian National University, Acton, ACT, Australia; University of Innsbruck, AUSTRIA

## Abstract

The use of diversity metrics has a long history in population ecology, while population genetic work has been dominated by variance-derived metrics instead, a technical gap that has slowed cross-communication between the fields. Interestingly, Rao’s Quadratic Entropy (RQE), comparing elements for ‘degrees of divergence’, was originally developed for population ecology, but has recently been deployed for evolutionary studies. We here translate RQE into a continuous diversity analogue, and then construct a multiply nested diversity partition for alleles, individuals, populations, and species, each component of which exhibits the behavior of proper diversity metrics, and then translate these components into [0,1]—scaled form. We also deploy non-parametric statistical tests of the among-stratum components and novel tests of the homogeneity of within-stratum diversity components at any hierarchical level. We then illustrate this new analysis with eight nSSR loci and a pair of close Australian marsupial (*Antechinus*) congeners, using both ‘different is different’ and ‘degree of difference’ distance metrics. The total diversity in the collection is larger than that within either species, but most of the within-species diversity is resident within single populations. The combined *A*. *agilis* collection exhibits more diversity than does the combined *A*. *stuartii* collection, possibly attributable to localized differences in either local ecological disturbance regimes or differential levels of population isolation. Beyond exhibiting different allelic compositions, the two congeners are becoming more divergent for the arrays of allele sizes they possess.

## Introduction

The use of genetic distance matrices to estimate genetic diversity within and among populations offers a number of benefits, including the ability to accommodate different genetic distance coding schemes, and computational tractability for large datasets. Here, we elaborate Rao’s Quadratic Entropy to quantify and statistically evaluate patterns of genetic diversity, both within and among strata of a multiply nested taxonomic hierarchy, which can be used for diverse types of genetic data. This approach is related to, but exhibits a variety of innovations, relative to the traditional variance-based criteria commonly applied in population genetics. Quadratic (*q* = 2) diversity metrics of several different types, originally developed for community ecology, have begun to infiltrate population genetic analysis, traditionally dominated by variance-based, least squares analyses [1–7]. To date, most such metrics have deployed ‘different is different’ coding of genetic markers, though sometimes measured along spanning networks [8–12], reflecting evolutionary separation. The use of ‘degree of difference’ as an evolutionary metric traces to early work [13–19], and has spawned recent efforts to elaborate diversity theory in that same vein [20–23].

In that context, Rao’s Quadratic Entropy (henceforth *Q*) has drawn some attention [24–29], because conversion into inverted Gini-Simpson 1/(1 − *Q*) form yields a well-behaved diversity metric, provided that certain conditions are met [30–35]. Our object here is to elaborate *Q*, incorporating the ‘degree of difference’ between pairs of individual genets into a well-behaved diversity metric. We can translate a considerable array of paired-individual Euclidean distance matrices, as deployed for Amova [8, 36–38], Permanova [39–41], or Gamova [42], into *Q*, and can then convert *Q* into diversity analogue that may prove evolutionarily and/or ecologically informative.

Conversion of *Q* into well-behaved diversity metric is only possible if [0 ≤ *Q* < 1]. There are three practical issues that must be dealt with in that translation. (1) Since quadratic genetic distance increases rapidly with the ‘degree of difference’, how are we to ensure that *Q* is properly bounded, given the wide array of quantitative divergence measures one could imagine for pairs of genets? (2) Can we estimate a well-behaved (and multiple level) partition of that total diversity, given the limited and typically unbalanced sampling routinely available from field studies? (3) Can we use this novel treatment for useful statistical evaluation of among-stratum diversification, as well as for evaluation of homo/heterogeneity of within-stratum diversity components? To illustrate both the formalisms and the utility of diversity translation, we employ a pair of Australian marsupial (*Antechinus*) congeners, sampled from contiguous Australian regions in New South Wales and Victoria, presenting evolutionary / geographic / environmental contrasts. We address a trio of additional questions: (4) How has evolutionary divergence within the complex been translated into genetic diversification within and between the two taxa? (5) Do responses to geographic or ecological challenges align with divergent patterns of diversity within the two organisms? (6) Do ‘different is different’ and ‘degree of difference’ treatments yield similar or disparate patterns of diversity within and between these close congeners?

## Mathematical and computational methods

### Rao’s quadratic entropy

We start with Rao’s quadratic entropy (henceforth *Q*), defined in terms of multi-locus genotypic arrays for the grand total collection of *N* diploid individuals. For any single genetic locus, we compute a squared allelic-pair distance as ‘0’ (if identical) or ‘1’ (if different) for all allelic pairs. Given a set of (*j*,*k* = 1,⋯,*J*) different alleles for that locus, we define *Q* for the total collection of *N* individuals (2*N* alleles) as:

$$Q=\sum_{j}\sum_{k}p_{j}∙p_{k}∙d_{jk}^{2},$$ (1)

where the relative frequencies of the *j*^*th*^ and *k*^*th*^ alleles are *p*_*j*_ and *p*_*k*_. Eq (1) can be rewritten in matrix form, using a matrix (**D**) of squared distances between all (4*N*^2^) allelic-pairs, and as a vector, ***P***′ = [(1/2*N*),⋯,(1/2*N*)], one entry per sampled allele

$$Q=P^{\prime }DP=(sumD/{4N}^{2}),$$ (2)

where *sum***D** is the total of (4*N*^2^) squared distances within matrix **D**, and *Q* is the average.

Many current genetic surveys deploy microsatellite or simple sequence repeat (SSR) markers, for which we routinely use ‘different is different’ coding, $\left(d_{jk}^{2}=0,\;same)\;or\;(d_{jk}^{2}=1,different\right)$, to construct a multi-allelic distance matrix **D**^C^ [8, 36–38]. For SSR markers, we can also translate differences in numbers of repeat units into a ‘degree of difference’ distance metric [43–45]. If the *j*^*th*^ and *k*^*th*^ alleles have *r*_*j*_ and *r*_*k*_ repeat units, respectively, then $d_{jk}^{2}={\left(r_{j}-r_{k}\right)}^{2}$, we can pack those squared repeat-number distances into a **D**^R^ matrix, connected by the assumption of single step mutation (SSM) models of evolution [14, 18–19]. We can use any quadratic Euclidean distance metric that makes sense for the problem at hand [46].

### Translating quadratic entropy into diversity

Provided that [0 ≤ *Q* < 1] for the total collection of all individuals, we can convert *Q* into a measure of total diversity within the collection, using inverted Gini-Simpson translation; for the *N* individuals (2*N* alleles), that translation takes the simple form

γ=1/(1−Q), (3)

where (γ) estimates the ‘effective number’ of equally frequent and equally different alleles within the collection. For any single locus of the ‘different is different’ **D**^C^ matrix, *Q* is properly bounded by virtue of its (0 vs 1) construction. For the ‘degree of difference’ **D**^R^ matrix, Eq (1) employs the squared difference in repeat units for the two alleles in question. Many individual elements in the matrix exceed ‘1’, and it is customary [31] to scale the pairwise distances to ensure that [0 ≤ *Q* < 1]. If the smallest allele for our single SSR locus has a count of (*r*_*min*_) repeat units and the largest has (*r*_*max*_) repeat units, each element of the single-locus **D**^R^ matrix should be divided by the maximum squared distance for that locus, $d_{max}^{2}={\left(r_{max}-r_{min}\right)}^{2}$, thus ensuring that [0 ≤ *Q* < 1]. To complete our scaling, we sum the squared distance values over loci, for either **D**^C^ or **D**^R^ loci, and then divide each multi-locus element by the number of loci (L) scored. The best scaling will depend on the genetic markers in question, of course, but by insuring that [0 ≤ *Q* < 1], we ensure that [1 ≤ γ < 2*N*] for diploids. We assume that our **D** matrices have been appropriately scaled, from the outset. The essential point is that the diversity translations of different distance matrices may shed useful light on the ecological and/or evolutionary reality we have sampled.

### The diversity partition

We are generally interested in a partition of that diversity across space, ecological context and/or taxonomic subdivision. To illustrate the partition of the total genetic diversity (γ) into separate within and among stratum levels of hierarchical sampling, consider a pair of congeneric species (S_A_ and S_B_). Let the numbers of sampled alleles within the respective species be (2*N*_A_ = 6, 2*N*_B_ = 4), and start with the example (five-allele array) illustrated in (Table 1). The average of all (4*N*^2^ = 100) elements in (**D**^C^) is *Q* = (*sum***D**^C^/4*N*^2^) = (78/100) = 0.78, from which Eq (3) yields (γ = 4.545) ‘effective (equi-frequent, equi-different) alleles’. The two species are not equally replicated within our sample, and we need to account for that imbalance with our estimation protocols. We compute a separate *Q*-value within each of the species, *Q*_WA_ = (22/36) and *Q*_WB_ = (10/16) and then convert the *Q*-values into separate diversity estimates within each of the species,

$$\sigma_{WA}=1/\left(1-Q_{WA}\right)=2.571\;and\;\sigma_{WB}=1/\left(1-Q_{WB}\right)=2.667,$$ (4)

within species A and B, respectively. We next compute a weighted average estimate of the within-species *Q*_WS_-value; for the sample entries in (Table 1), the sample size weights are

$$f_{WA}={4N}_{WA}^{2}/({4N}_{WA}^{2}+{4N}_{WB}^{2})\;f_{WB}={4N}_{WB}^{2}/\left({4N}_{WA}^{2}+{4N}_{WB}^{2}\right),$$ (5)

yielding weighted average within-species (*Q*_WS_) and diversity (σ_WS_) values of the form

$$Q_{WS}=[f_{WA}∙Q_{WA}+f_{WB}∙Q_{WB}]=(8/13)\;\sigma_{WS}=1/\left(1-Q_{WS}\right)=2.6.$$ (6)

The extension to multiple, unequally sampled species, is obvious. We can also compute an among-species *Q*_AS_ value and (derivative) diversity (δ_AS_) estimate, which is the ‘effective (equi-frequent, equi-different) number’ of species with no cross-species allelic sharing. To illustrate that extraction, in the context of (Table 1), we compute

$$\delta_{AS}=(\gamma /\sigma_{WS})=1/(1-Q_{AS})=\left(1-Q_{WS}\right)/(1-Q)=1.748,$$ (7)

which we can back-translate into an equivalent ‘among-species’ (*Q*_AS_) value,

$$Q_{AS}=\left(Q-Q_{WS}\right)/\left(1-Q_{WS}\right)=\left(\delta_{AS}-1\right)/\delta_{AS}=0.428.$$ (8)

By construction, γ = (δ_AS_ ∙ σ_WS_) = (1.748) ∙ (2.6) = 4.545 for the example (Table 1).

**Table 1:** Illustrative distance matrix (D^C^) for two species: (A, three diploid individuals) and (B, two diploid individuals): five different alleles (g-1) · · · (g-5), with squared distances $\left(d_{jk}^{2}=0\right)$ if alleles are identical but $\left(d_{jk}^{2}=1\right)$ if different.

	Species A						Species B				
Allele	g-1	g-1	g-1	g-2	g-2	g-3	g-3	g-4	g-4	g-5	Allele
g-1	0	0	0	1	1	1	1	1	1	1	g-1
g-1	0	0	0	1	1	1	1	1	1	1	g-1
g-1	0	0	0	1	1	1	1	1	1	1	g-1
g-2	1	1	1	0	0	1	1	1	1	1	g-2
g-2	1	1	1	0	0	1	1	1	1	1	g-2
g-3	1	1	1	1	1	0	0	1	1	1	g-3
g-3	1	1	1	1	1	0	0	1	1	1	g-3
g-4	1	1	1	1	1	1	1	0	0	1	g-4
g-4	1	1	1	1	1	1	1	0	0	1	g-4
g-5	1	1	1	1	1	1	1	1	1	0	g-5
Allele	g-1	g-1	g-1	g-2	g-2	g-3	g-3	g-4	g-4	g-5	Allele
	Species A						Species B				

Similar treatment of the (sample frame determined) max-diversity dataset (Table 2) yields maximum achievable (Q*) values and their derivative diversity maxima, using the same computational stream as used in Eqs (3–8). Both the observed (Table 1) *Q*-values and their sample-frame dependent (Table 2) maximum Q*-values are presented for the exemplar in Table 3, where we also present a set of [0,1]-scaled diversity estimates, which we will elucidate below.

**Table 2:** Maximum distance matrix (D^C^) for two species: (A, three diploid individuals) and (B, two diploid individuals, with ten different alleles (g-1) · · · (g-10); squared distances $\left(d_{jk}^{2}=0\right)$ if alleles are identical but $\left(d_{jk}^{2}=1\right)$ if different; analysis yields sample-frame dependent maximum Q*-values and their translations into maximum diversity estimates.

	Species A						Species B				
Allele	g-1	g-2	g-3	g-4	g-5	g-6	g-7	g-8	g-9	g-10	Allele
g-1	0	1	1	1	1	1	1	1	1	1	g-1
g-2	1	0	1	1	1	1	1	1	1	1	g-2
g-3	1	1	0	1	1	1	1	1	1	1	g-3
g-4	1	1	1	0	1	1	1	1	1	1	g-4
g-5	1	1	1	1	0	1	1	1	1	1	g-5
g-6	1	1	1	1	1	0	1	1	1	1	g-6
g-7	1	1	1	1	1	1	0	1	1	1	g-7
g-8	1	1	1	1	1	1	1	0	1	1	g-8
g-9	1	1	1	1	1	1	1	1	0	1	g-9
g-10	1	1	1	1	1	1	1	1	1	0	g-10
Allele	g-1	g-2	g-3	g-4	g-5	g-6	g-7	g-8	g-9	g-10	Allele
	Species A						Species B				

**Table 3:** Observed *Q*-values for exemplar (Table 1) and maximum *Q*-values (Table 2), translated into observed, maximum, and [0,1]-scaled estimates for total diversity, a separate within-species estimate for each species, a weighted average within-species estimate, and an among-species estimate.

Computed	Total	Among	AveWithin	Within	Within
Criterion	Study	Species	Species	Species A	Species B
Data Diversity Estimate	4.545	1.748	2.600	2.571	2.667
Data *Q*-Value	0.780	0.428	0.615	0.611	0.625
[0,1]- Scaled Diversity	0.867	0.892	0.762	0.733	0.833
Max Possible *Q*-Value	0.900	0.480	0.808	0.833	0.750
Max Diversity Possible	10.000	1.923	5.200	6.000	4.000

### Extending the diversity partition downward

We typically sample multiple populations within each of our sample species, and for diploids (and also polyploids) we may also want to elaborate diversity within and among single individuals. For both purposes, we need to extend our diversity partition downward, so imagine six (6) populations, two (2) from species A and four (4) from species B. We construct a separate distance matrix within each population, extract their within-population (*Q* and Q*) values, and convert those into separate diversity estimates for each population,

$$\alpha_{WP1}=1/\left(1-Q_{WP1}\right)\;\cdots \;\alpha_{WP6}=1/\left(1-Q_{WP6}\right)$$ (9)

Using the same strategy as for Eqs (5) and (6), we compute a (within-population) average *Q*_WP_ value for two populations in Species A and the four populations in Species B, using quadratic sample-size weights of the form:

$$\begin{array}{l} \;f_{P1}=N_{P1}^{2}/\left(N_{P1}^{2}+N_{P2}^{2}\right)\;f_{P2}=N_{P2}^{2}/\left(N_{P1}^{2}+N_{P2}^{2}\right),\\ \mathrm{and}\\ \;f_{P3}=N_{P3}^{2}/\left(N_{P3}^{2}+N_{P4}^{2}+N_{P5}^{2}+N_{P6}^{2}\right)\;\cdots \;f_{P6}=N_{P6}^{2}/\left(N_{P3}^{2}+N_{P4}^{2}+N_{P5}^{2}+N_{P6}^{2}\right). \end{array}$$ (10)

We then translate those weighted-average (*Q*_WP_) values into separate within-population diversity estimates for Species A and B, respectively,

$$\alpha_{WPA}=1/(1-Q_{WPA})\;\alpha_{WPB}=1/\left(1-Q_{WPB}\right).$$ (11)

If we average all six within-population *Q*-values (weighted by their respective quadratic sample sizes), we obtain an average (*Q*_WP_) for the whole study, and can translate that into a study-wide average estimate of the within-population diversity,

$$\alpha_{WP}=1/\left(1-Q_{WP}\right).$$ (12)

We are not constrained to balanced sampling at any level, but we do need to account explicitly for whatever sampling imbalance exists within the dataset.

For diploids, we can also extract estimates of within-individual (among-allele) diversity for each locus, as each individual is represented by a (2 x 2) submatrix (Table 1 and Table 2); we have *N* such sub-matrices for the study. We later illustrate this new analysis for a pair of strongly outbred species [47], where subdivision of within-individual diversity is not very helpful. For organisms showing non-random mating systems, however, extracting sub-individual diversity components may prove valuable, and we describe their estimation in S1 Appendix.

Given that our populations are nested within species, we also need to compute traditional among-population (*Q*_AP_) and $\left(Q_{AP}^{*}\right)$ values and to translate those into both estimated (β_AP_) and maximum possible $\left(\beta_{AP}^{*}\right)$ diversity. By analogy with Eq (7), we deploy

$$\beta_{AP}=(\sigma_{WS}/\alpha_{WP})=(1-Q_{WP})/\left(1-Q_{WS}\right),$$ (13)

and noting that β_AP_ = 1/(1−*Q*_AP_), we back-translate Eq (13) to extract

$$Q_{AP}=\left(Q_{WS}-Q_{WP}\right)/\left(1-Q_{WP}\right).$$ (14)

With similar definition and estimation of the within-individual diversity (ω_WI_) and the among-individual diversity (ε_AI_), both nested within single populations (S1 Appendix), we have now defined and elaborated (an RQE-derivative) diversity estimation cascade that elaborates the traditional three-level panoply into a multiplicative multi-level cascade,

$$\gamma =(\omega_{WI}∙\varepsilon_{AI}∙\beta_{AP}∙\delta_{AS})$$ (15)

### Diversity desiderata

Beyond sheer definition and estimation, well-behaved diversity components should exhibit a set of key features. The within-stratum components represent ‘effective numbers’ of (equi-frequent and equi-different) alleles within each level of the nested hierarchy, and these within-stratum estimates should satisfy the condition,

$$1\leq \omega_{WI}\leq \alpha_{WP}\leq \sigma_{WS}\leq \gamma \leq 2N,$$ (16)

which our estimates do. The among-stratum components represent ‘effective numbers’ of non-overlapping allelic collections for (equi-frequent and equi-different) sub-strata: among individuals of a single population, among populations within a single species, and among species of the total collection. These among-stratum components are explicitly defined so that

$$\alpha_{WP}=(\varepsilon_{AI})∙(\omega_{WI})\;\sigma_{WS}=(\beta_{AP})∙(\alpha_{WP})\;\gamma =(\delta_{AS})∙(\sigma_{WS}).$$ (17)

Given strict nesting of the *Q*-values, and within the constraints of Eqs (7, 8, 13 and 14), all of the components are free to vary independently. Our within- and among stratum diversity estimates meet all of those conditions.

If we add genetic variety at any level, diversity must increase. Consider a single population (P_1_), nested within a species (S_A_). For **D**^C^ coding, if any existing allele (within that population) is replaced by a novel allele (for that population), the within-population diversity (α_WP1_) will increase. That also increases the within-species diversity (σ_WSA_) of the species, within which (P_1_) is nested. If other populations within (S_A_) show some genetic overlap but do not have this novel variant, the among-populations diversity will also increase. For **D**^R^ coding, ‘degree of difference’ also matters, and if the novel variant in (P_1_) is beyond the ‘size range’ of previously represented alleles in (P_1_), the internal diversity (α_WP1_) of that population will increase. If it is also beyond the size range of the species (S_A_), within which it is nested, so will be (σ_WS_) and (β_AP_), etc. Our estimation protocols ensure that all of our diversity estimates meet the desiderata.

### Maximum and [0,1]—scaled diversity

Without scaling, a minimum achievable diversity estimate at any level is ‘1’ by construction. If we were to compute the diversity cascade, achievable from a (2*N* x 2*N*) matrix with every off-diagonal element being identically ‘1’, our diversity components would attain (sample-frame constrained) maximum values (Table 2). That would maximize all the *Q*-values and their diversity translations. It is usual to estimate and compare diversity metrics from (modest and typically unbalanced) samples, and it is often useful to gauge those estimates, relative to the minima and maxima achievable, given the sampling limitations. We will henceforth denote the max-diversity distance matrix (and all of its derivate summations, *Q*-values, and diversity transforms) with an (*). If all genets (at any level) are equally different and represented once each, the maximum diversity values become the raw numbers of those elements. The most diverse collection attainable has 2*N* equally different alleles. A pair of equally sampled species, sharing no alleles in common, yields $\left(\delta_{AS}^{*}=2\right)$, while a trio of equally sampled populations, sharing no alleles in common, yields $\left(\beta_{AP}^{*}=3\right)$, etc. With unbalanced sampling, those maxima are reduced, but whether sampling is balanced or not, all diversity estimates are explicitly (sample frame) bounded, both above and below,

$$\begin{array}{l} \;1\leq \gamma \leq \gamma^{*}\;1\leq \sigma_{\mathrm{WS}}\leq \sigma_{\mathrm{WS}}^{*}\;1\leq \alpha_{\mathrm{WP}}\leq \alpha_{\mathrm{WP}}^{*}\;1\leq \omega_{\mathrm{WI}}\leq \omega_{\mathrm{WI}}^{*},\\ \mathrm{and}\\ \;1\leq \delta_{\mathrm{AS}}\leq \delta_{\mathrm{AS}}^{*}\;1\leq \beta_{\mathrm{AP}}\leq \beta_{\mathrm{AP}}^{*}\;1\leq \varepsilon_{\mathrm{AI}}\leq \varepsilon_{\mathrm{AI}}^{*}. \end{array}$$ (18)

We can scale an estimate of shared diversity among strata at any given level, ranging from 0 (no sharing) to 1 (complete sharing and identical frequencies of) all elements [20]. Starting from that criterion, we can define a complementary estimate of non-overlap, ranging from 0 (total sharing and identical frequencies of elements) to 1 (no sharing of elements). For the RQE-derivative diversity metrics above, that translation yields a remarkably convenient and easily computed set of [0,1]—scaled diversity estimates [48] (S2 Appendix),

$$\begin{array}{l} \;\gamma^{∼}=\left(Q/Q^{*}\right)\;\left(\sigma_{\mathrm{WS}}^{∼}\right)=\left(Q_{\mathrm{WS}}/Q_{\mathrm{WS}}^{*}\right)\;\left(\sigma_{\mathrm{WS}}^{∼}\right)=\left(Q_{\mathrm{WS}}/Q_{\mathrm{WS}}^{*}\right)\;\left(\omega_{\mathrm{WI}}^{∼}\right)=\left(Q_{\mathrm{WI}}/Q_{\mathrm{WI}}^{*}\right),\\ \mathrm{and}\\ \;\left(\delta_{\mathrm{AS}}^{∼}\right)=\left(Q_{\mathrm{AS}}/Q_{\mathrm{AS}}^{*}\right)\;\left(\beta_{\mathrm{AP}}^{∼}\right)=\left(Q_{\mathrm{AP}}/Q_{\mathrm{AP}}^{*}\right)\;\left(\varepsilon_{\mathrm{AI}}^{∼}\right)=\left(Q_{\mathrm{AI}}/Q_{\mathrm{AI}}^{*}\right). \end{array}$$ (19)

Returnning to our example array (Table 1 and Table 2), the [0,1]-scaled diversity estimates (third line) in (Table 3), are obtained by computing the corresponding ratios of the data *Q*-estimates from the line just above and the (Q*) maxima from the line just below. For Table 3, we compute (γ^∼^ = (0.780/0.900) = 0.867), and similarly, for the other estimates. Each element of Eq (19) is thus explicitly [0,1]-scaled for the sampling frame itself. Such [0,1]—scaling provides a useful sense of ‘how large or small the diversity is’ at any given level, relative to ‘how large or small it cold be’, given the sampling frame. The translation is that ‘0’ represents no genetic diversification of the elements under consideration, and that ‘1’ represents maximum achievable diversification (no overlap), given the sampling frame.

### Statistical inference on diversity components

By recasting the RQE argument in distance matrix form, we can convert any (genetically sensible) Euclidean (positive semi-definite) inter-allelic distance matrix **D** for the *N* individuals (2*N* alleles per locus) into a nested cascade of estimated diversity components. Beyond estimation, we can (and should) assess the statistical credibility of whatever we estimate. The total diversity provides a system-wide baseline, but since we would not conduct the exercise in the absence of meaningful genetic diversity, a test of whether (γ^∼^) > 0 would be rather pointless. A more interesting set of questions would be whether there is credible diversity among species $(\delta_{AS}^{∼})>0$ or diversity among populations $(\beta_{AP}^{∼})>0$ within them, or even (in some cases) whether there is diversity among individuals $(\varepsilon_{AI}^{∼})>0\;$ within the same population. The traditional variance-derivative tests of inter-population divergence, such as (*F*_ST_) and (*G*_ST_), have been challenged as poorly bounded, but alternative criteria that are [0,1]-scaled and better-behaved have been offered. We show (S2 Appendix) that [0,1]—scaled among-population diversity $\left(\beta_{AP}^{∼}\right)$ is an extension of Jost’s (*D*) criterion [49–50] to the more general (unbalanced sampling) case. We extend that treatment upward to the among-species $\left(\delta_{AS}^{∼}\right)$ and downward to the among-individual $\left(\varepsilon_{AI}^{∼}\right)$ levels of scaled diversity estimates, both appropriately bounded and well behaved.

We might also find it useful to test whether: (a) the separate within-species diversity estimates $\left(\sigma_{WS}^{∼}\right)$ are credibly homogeneous from species to species, or (b) whether the within-population diversity estimates $\left(\alpha_{WP}^{∼}\right)$ are credibly homogeneous from population to population (within, or even among species), or even (c) whether the within-individual diversity estimates $\left(\omega_{WI}^{∼}\right)$ are credibly homogeneous among individuals, within or among populations or species. We show (S3 Appendix) that a test of the hypothesis of homogeneous within species diversity values is tantamount to a Bartlett’s test [51] of homogeneity of the corresponding within-species variances. The same equivalence applies at the within-population and within-individual levels. Failure of any of our within-stratum homogeneity tests would provide signals of differential demographic, ecological and/or evolutionary pressures that have shaped such diversity in different fashions or to different degrees within different sampling strata.

Normal or multinomial statistical theory assumptions are too restrictive for the wide array of data sets and contextual situations under real world consideration, so we deploy here a set of non-parametric test criteria, with (locus by locus) permutation of alleles among the strata under consideration, while holding the realized sampling frame constant (S2 and S3 Appendices). These estimation and testing protocols are embedded within the QDiver routine, now available within GenAlEx 6.51 (http://biology.anu.edu.au/GenAlEx/; [52–53]).

### Diversity analysis of paired *Antechinus* congeners

Here we illustrate these new tools with the Australasian marsupial genus *Antechinus*, comprised of small ground-dwelling and climbing predators of forests, woodlands and heathlands. Morphological and phylogenetic research in recent decades has identified several previously unrecognized species-level splits within the genus. Both *A*. *stuartii* and *A*. *agilis* were once viewed as a single species (*A*. *stuartii*), but recent research has indicated that they are separate species, with a geographic break approximately 200 km south of Sydney, New South Wales [54]. Based on nuclear (*IRBP*, *RAG*1, *bFib*7) and mitochondrial (*cyt-b*, 12sRNA, 16sRNA) sequence analysis [55], these congeners are thought to have diverged in the early Pliocene. Much of the published life history research on *Antechinus* was conducted within the range of *A*. *agilis*, predating recognition of two species, but *A*. *stuartii* is demographically quite similar. Both are small, semelparous carnivores (approx. 15-50g); polyandrous females give birth to (6–10) offspring (there is geographic variation in teat number) each spring. Most females die after weaning their first clutch, but a few survive to breed in a second year. The males die shortly after an intense breeding season in their second year.

### Regional allopatry

**Fig 1:**
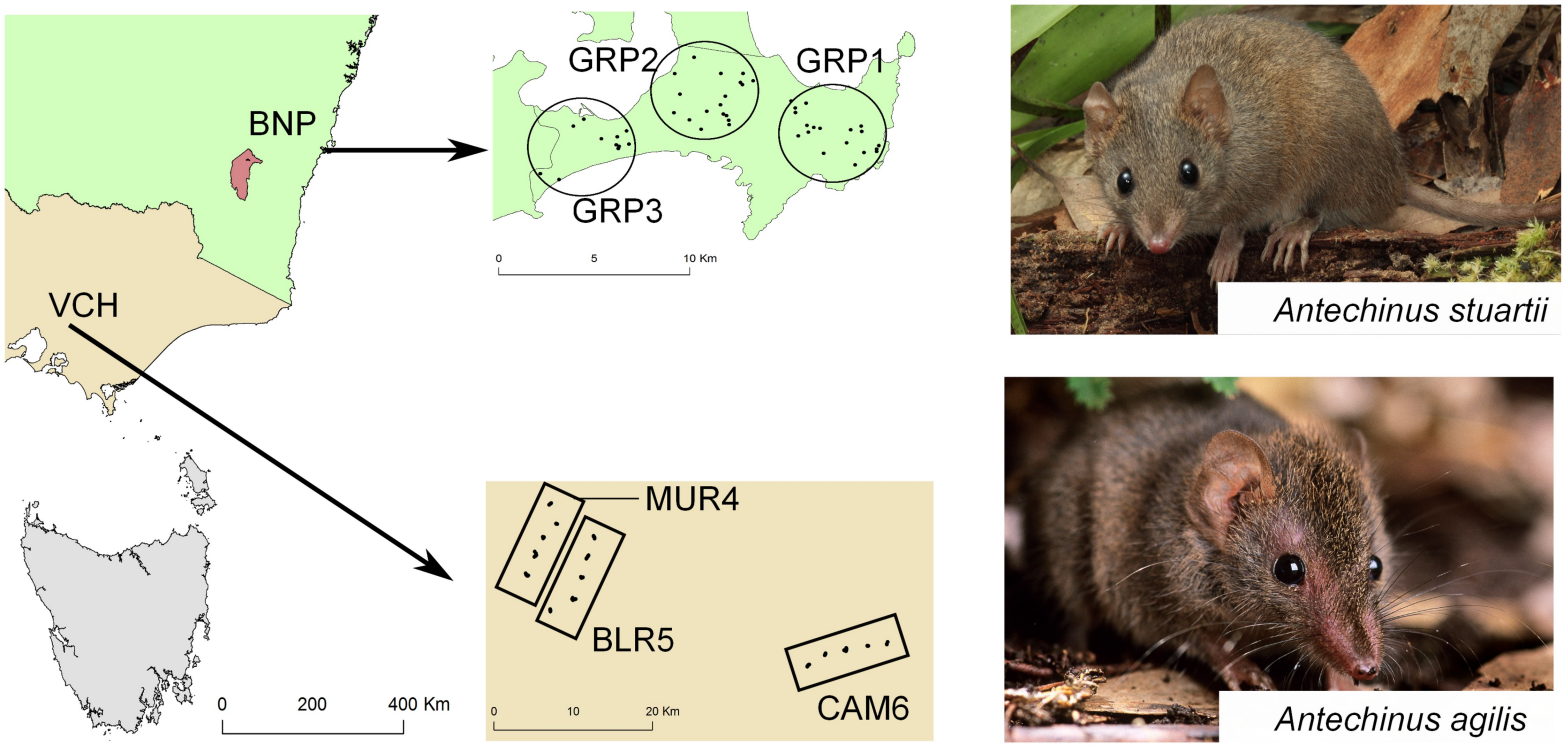
Map of the study locations for *Antechinus stuartii* (Booderee National Park, New South Wales) and *A*. *agilis* (Victoria Central Highlands, Victoria), Australia. Photo credits go to Stephen Mahony and Esther Beaton, respectively.

We sampled *A*. *stuartii* from Booderee National Park (BNP) in New South Wales and *A*. *agilis* from the Victoria Central Highlands (VCH) in Victoria (Banks *et al*. 2011), separated by about 500 km (Fig 1). Within each species, sampling involved a trio of spatially separated trapping areas, each treated here as a separate ‘population’ for illustrative purposes (Fig 1). Each species is common within its own range, and there are no overt habitat discontinuities or overt barriers to gene flow, barring the effects of dispersal distance itself. BNP populations are spread out along a peninsula, with GRP1 most seaward (and most constrained), about twice as far from GRP3 (the most landward population) as it is to GRP2. For VCH populations, CAM6 is about four times as far from BLR5 as is the latter from MUR4. The population samples themselves are more widely separated for *A*. *agilis* (VCH) than for *A*. *stuartii* (BNP). The average pre-mating dispersal distance of males is over 1000 m, while that for females averages less than 100 m [56–58]. Genetic isolation (over 10s of km) may well impact our within-species decomposition (σ_WS_ = β_AP_ **·** α_WP_) for these organisms.

### Nuclear microsatellite markers

**Fig 2:**
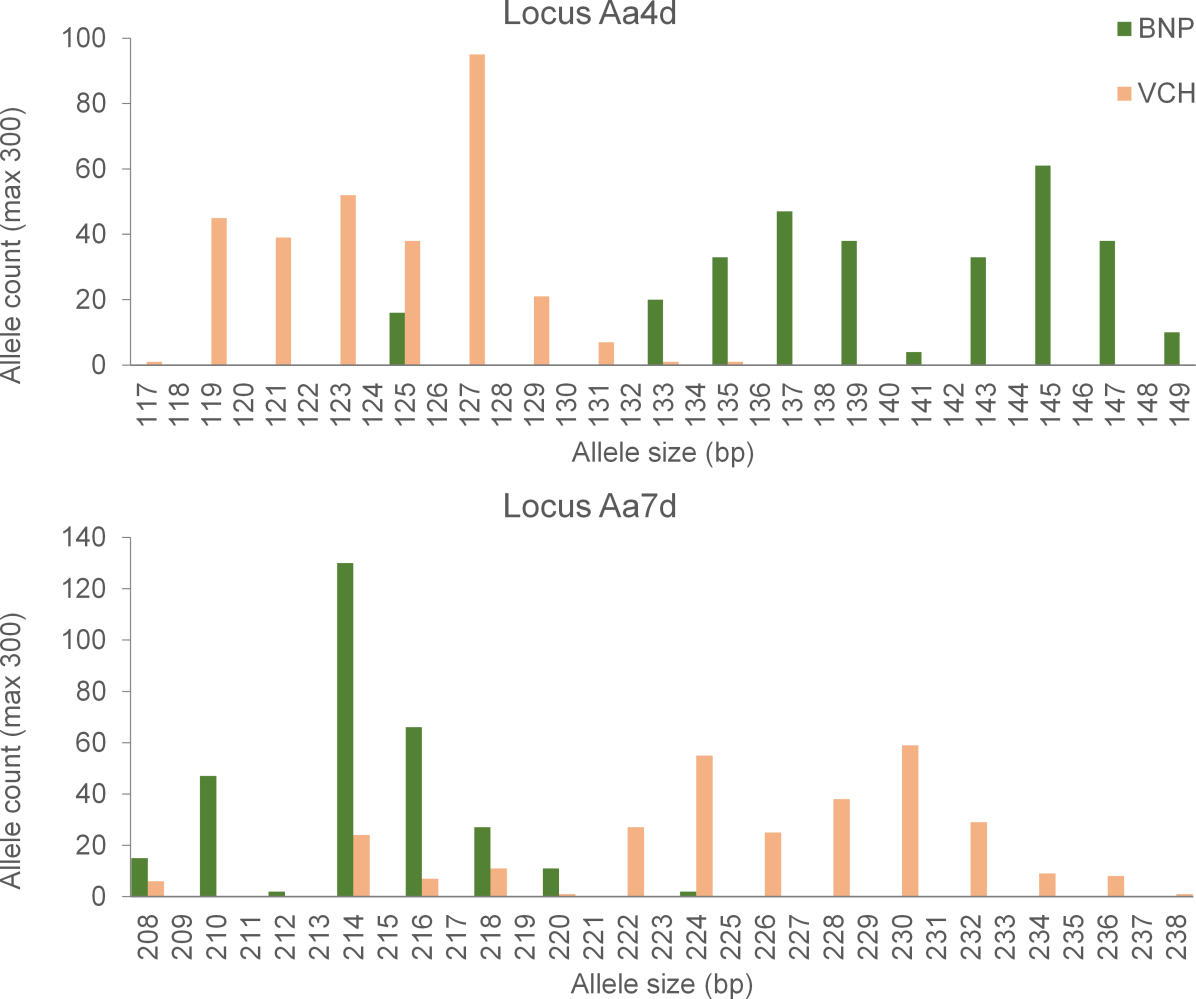
A pair of typical two-nucleotide step SSRs (Aa4d and Aa7d) for *Antechinus stuartii* (in Booderee National Park (BNP), New South Wales) and *A*. *agilis* (in Victoria Central highlands (VCH), Victoria) Australia.

For this study, we have analyzed eight nuclear SSR loci (Aa7d, Aa2e, Aa2g, Aa4d, Aa7f, Aa7m, Aa4k, Aa2b) for each of 50 individuals for each population, a grand total of (2*N* = 600) alleles for each locus (SSR lab protocols in S4 Appendix). The loci used here are a subset of those previously assayed for these species, filtered here for an absence of null-alleles, as well as conformance to regular allele step size criteria [57]. The regularity restriction was applied to remove a trio of loci with a high proportion of allelic step sizes that were less than the length of the microsatellite repeat motif itself. We illustrate with a pair of allele size distributions (Aa4d and Aa7d), each with a two-nucleotide repeat motif, illustrating non-trivial ‘ladder offset’ between the allelic batteries of the two species, in spite of some allelic overlap and sharing (Fig 2).

### Different is different (D^C^) and degree of difference (D^R^) coding

We used traditional (0 vs 1) coding for our (**D**^C^) treatment (Table 1), but for each of the eight loci separately, then added the eight matrices for all loci, element by element, as is standard for multi-locus distance analysis. We then divided each element in the multi-locus **D**^C^ matrix by (L = 8), reducing each matrix to (average) single-locus form and ensuring that [0 ≤ *Q* < 1], so that all **D**^C^—derived diversity elements are properly bounded and well behaved. The two species occupy somewhat different (though overlapping) sectors of the eight nSSR ladders (Fig 2), so for each locus of our **D**^R^ matrix, we computed the squared distance between any pair of alleles as the square of the number of (two-nucleotide) steps between them, divided by the maximum squared distance $\left(d_{max}^{2}\right)$ for that locus. Finally, we added the eight single-locus matrices together, and we divided each element of that summation by (L = 8), ensuring that all **D**^R^—derived diversity components are properly bounded and well-behaved. This (*R*_ST_) ‘degree of difference’ metric is convenient, but not the only possible choice, a matter to which we will return in the **Discussion**.

### Diversity within populations

For each of the six sampled populations, we present the [0,1]—scaled values $\left(\alpha_{WP}^{∼}\right)$, as well as separate species averages and a pooled study-wide average for both **D**^C^and **D**^R^ coding (Table 4). The geographically most isolated populations have less internal diversity within both species, $\left(\alpha_{P1}^{∼}<\alpha_{P2}^{∼}<\alpha_{P3}^{∼}\right)$ within *A*. *stuartii* (BNP) and $\left(\alpha_{P4}^{∼}>\alpha_{P5}^{∼}>\alpha_{P6}^{∼}\right)$ within *A*. *agilis* (VCH). The first of these (involving peninsular GRP1) is marginally significant; the second (simply more isolated CAM6) is not. The within-population components for *A*. *agilis* (VCH) are about twice as large as those for *A*. *stuartii* (BNP) for either **D**^C^ or **D**^R^ coding. Having scaled **D**^C^ elements by $(d_{max}^{2})=1$ and those for **D**^R^ by $(d_{max}^{2})=275$, all of our within-population components are an order of magnitude smaller for **D**^R^ than they are for **D**^C^ coding (Table 4).

**Table 4:** Scaled within-population allelic diversity $\left(\alpha_{WP}^{∼}\right)$ for both D^C^ and D^R^ coding within *Antechinus stuartii* (Booderee National Park), within *A*. *agilis* (Victoria Central Highlands), and within the entire study, with Bartlett’s tests of within-population homogeneity.

*Antechinus stuartii* (BNP)			**D**^C^ Coding	*Antechinus agilis* (VCH)		
GRP1	GRP2	GRP3	[0,1]—Scaled Diversity	MUR4	BRL5	CAM6
0.435	0.453	0.476	$(\alpha_{WP}^{∼})\;$ Estimates	0.841	0.836	0.831
Average & Bartlett’s Test			Average & Bartlett’s Test	Average & Bartlett’s Test		
$\alpha_{WP}^{∼}\;=\;0.455\;(P<0.041)$			$\alpha_{WP}^{∼}=0.645\;(P<0.001)$	$\alpha_{WP}^{∼}=0.836\;(P>0.64)$		
*Antechinus stuartii* (BNP)			**D**^R^ Coding	*Antechinus agilis* (VCH)		
GRP1	GRP2	GRP3	[0,1]—Scaled Diversity	MUR4	BRL5	CAM6
0.031	0.036	0.041	$(\alpha_{WP}^{∼})\;$ Estimates	0.072	0.073	0.069
Average & Bartlett’s Test			Average & Bartlett’s Test	Average & Bartlett’s Test		
$\alpha_{WP}^{∼}\;=\;0.036\;(P<0.07)$			$\alpha_{WP}^{∼}=0.054\;(P<0.001)$	$\alpha_{WP}^{∼}=0.071\;(P>087)$		

### Partitioning diversity along the taxonomic hierarchy

Study-wide (and within-species) diversity cascades are presented for both **D**^C^ and **D**^R^ coding in (Table 5). The total γ^∼^ = (*Q*/Q*) and among-species $\left(\delta_{AS}^{∼})=(Q_{AS}/Q_{AS}^{*}\right)$ estimates are explicitly defined for the two species jointly, but we also define within-species $\left(\sigma_{WS}^{∼})=(Q_{WS}/Q_{WS}^{*}\right)$ and among-population $\left(\beta_{AP}^{∼})=(Q_{AP}/Q_{AP}^{*}\right)$ components for each of the species separately, to augment the average within-population $\left(\alpha_{WP}^{∼})=(Q_{WP}/Q_{WP}^{*}\right)$ diversities within those same species, the latter drawn from (Table 4). Total diversity is the product of all components in the multiply-nested cascade, averaged over 600 alleles for each of (L = 8) nSSR loci. It is large for both **D**^C^ (γ^∼^ = 0.807) and for **D**^R^ (γ^∼^ = 0.125) coding, but reflects the scaling difference between the two coding schemes.

**Table 5:** Scaled diversity values for both D^C^ and D^R^ coding, and for both *Antechinus stuartii* and *A*. *agilis*: study total (γ^∼^), among-species $\left(\delta_{AS}^{∼}\right)$, within-species $\left(\sigma_{WS}^{∼}\right)$, among-populations $\left(\beta_{AP}^{∼}\right)$, and within-populations $\left(\alpha_{WP}^{∼}\right)$, with Bartlett’s homogeneity tests of the within stratum components.

*Antechinus stuartii* (BNP)	**D**^C^ Study-Wide Average	*Antechinus agilis* (VCH)
Booderee National Park	Diversity & Statistical Tests	Victoria Central Highlands
Diversity Components	γ^∼^ = 0.807	Diversity Components
& Statistical Tests	$\delta_{AS}^{∼}=0.891\;(P<0.001)$	& Statistical Tests
$\sigma_{WS}^{∼}=0.461$	$\sigma_{WS}^{∼}=0.651\;(P<0.001)$	$\sigma_{WS}^{∼}=0.841$
$\beta_{AP}^{∼}=0.025\;(P<0.001)$	$\beta_{AP}^{∼}=0.040\;(P<0.001)$	$\beta_{AP}^{∼}=0.088\;(P<0.001)$
$\alpha_{WP}^{∼}=0.455\;(P<0.041)$	$\alpha_{WP}^{∼}=0.645\;(P<0.001)$	$\alpha_{WP}^{∼}=0.836\;(P>0.64)$
*Antechinus stuartii* (BNP)	**D**^R^ Study-Wide Average	*Antechinus agilis* (VCH)
Booderee National Park	Diversity & Statistical Tests	Victoria Central Highlands
Diversity Components	γ^∼^ = 0.125	Diversity Components
& Statistical Tests	$\delta_{AS}^{∼}=0.150\;(P<0.001)$	& Statistical Tests
$\sigma_{WS}^{∼}=0.036$	$\sigma_{WS}^{∼}=0.054\;(P<0.001)$	$\sigma_{WS}^{∼}=0.071$
$\beta_{AP}^{∼}=0.001\;(P>0.90)$	$\beta_{AP}^{∼}=0.001$	$\beta_{AP}^{∼}=0.001\;(P>0.74)$
$\alpha_{WP}^{∼}=0.036\;(P>0.07)$	$\alpha_{WP}^{∼}=0.053\;(P<0.001)$	$\alpha_{WP}^{∼}=0.071\;(P>0.87)$

These two species are substantially divergent, with $\left(\delta_{AS}^{∼}=0.891,P<0.001\right)$ for **D**^C^ coding and $\left(\delta_{AS}^{∼}=0.150,P<0.001\right)$ for **D**^R^ coding, signatures of phylogenetic diversification for congeners separated since the early Pliocene [55]. Both of the within-species diversity components are substantial, but that within *A*. *agilis* (VCH) is about twice as large as that within *A*. *stuartii* (BNP), both for **D**^C^ $\left(\sigma_{WS-VCH}^{∼}=0.841>\sigma_{WS-BNP}^{∼}=0.461,P<0.001\right)$ and for **D**^R^ $\left(\sigma_{WS-VCH}^{∼}=0.071>\sigma_{WS-BNP}^{∼}=0.036,P\leq 0.001\right)$ coding.

Neither regional landscape shows any overt barriers to gene flow, but the dispersal challenge posed by sheer distance is greater for *A*. *agilis* (VCH) than for *A*. *stuartii* (BNP) populations (Fig 1). Given the greater isolation of CAM6 from (BLR5 and MUR4) than of GRP1 from (GRP2 and GRP3), we might anticipate greater among-population diversity within *A*. *agilis* (VCH) than within *A*. *stuartii* (BNP). As anticipated, **D**^C^ coding yields the expected pattern $\left(\beta_{AP-VCH}^{∼}=0.088,P\leq 0.001\right)$ and $\left(\beta_{AP-BNP}^{∼}=0.025,P\leq 0.001\right)$, but population subdivision is virtually nil $\left(\beta_{AP-VCH}^{∼}=0.001=\beta_{AP-BNP}^{∼}\right)$ within either species for **D**^R^ coding. Populations diverge somewhat for allelic composition within either species, but with no net ‘ladder offset’ among those populations. Given the deep phylogenetic history of this genus, the two taxa should be substantially more diverse at the species level than at the population level (within either of them), and that is what we find. While the among-species $\left(\sigma_{AS}^{∼}\right)$ component is an order of magnitude larger than the among-population $\left(\beta_{AP}^{∼}\right)$ component for ‘different is different’ (**D**^C^) coding, however, it is two orders of magnitude larger for ‘degree of difference’ (**D**^R^) coding. The nSSR ‘ladder offset’ between these congeners (Fig 2) constitutes a compelling diversity signature of long-term evolutionary separation.

## Discussion

### Overview of outcomes

We have elaborated a classic approach for estimating (*q* = 2) genetic diversity metrics that meet the standard desiderata of diversity measures. We first defined pairwise genetic distances between all pairs of (2*N*) alleles for each genetic locus, and packed those into a square distance matrix, using both **D**^C^ and **D**^R^ coding schemes. We then divided each element by the largest in the matrix $\left(d_{max}^{2}\right)$, extracted a bounded form of Rao’s quadratic entropy [0 ≤ *Q* < 1], and converted that to a measure of diversity for the whole collection (γ). We extended the treatment to a multiply-nested partition of the total diversity for the general case of unbalanced sampling, top to bottom of the hierarchy. We scaled each of the diversity components [0,1], using sample frame restrictions. Finally, we deployed tests for the among-species, among-population, and among-individual components, as well as novel homogeneity tests for the within-stratum components. All of these innovations are now encoded within the QDiver routine of GenAlEx 6.51 (http://biology.anu.edu.au/GenAlEx/; [52–53]).

We illustrated this new analysis with two *Antechinus* congeners (*A*. *stuartii* and *A*. *agilis*), using eight nSSR loci, treated in both (**D**^C^) and (**D**^R^) fashion. There is large (phyletic) diversity between the two species, but about twice as much diversity within *A*. *agilis* as within *A*. *stuarti*. The ‘population structure’ within *A*. *agilis* was also greater than that within *A*. *stuartii*. There are two possible explanations: (a) greater frequency of disturbance (wildfire) for BNP (*A*. *stuartii*) than for VCH (*A*. *agilis*), which could induce local bottlenecks and slow recovery of local population panmixis [59–61]; and (b) greater spatial dispersion of sampling sites within VCH than within BNP. There is some confounding of regional fire history with regional spatial separation here, but the spatial dispersion differences seem the more likely explanation. Finally, the ratio of among-species to among-populations diversity was an order of magnitude larger for **D**^R^ than for **D**^C^ coding, with minor allele frequency divergence (but no ladder shifts) among populations within either species, coupled with major ladder shifts between the two species.

### Scaling considerations

We have here deployed standard ‘degree of difference’ (*R*_*ST*_) coding for the **D**^R^ treatment. Beyond some level of phylogenetic separation, however, the use of **D**^R^ coding may not be linear with phylogenetic time, given the inherent mutational homoplasy of microsatellite substitution [62]. Particularly with small sample sizes, small numbers of SSR loci, and deep time depth, it is possible to under-estimate divergence with classic (*R*_ST_) scaling, and that estimation error increases with evolutionary time. Various workers have suggested using negative binomial coding [43, 45, 63–64], for which ‘degree of difference’ scaling is log-linear (rather than linear) with increasing phylogenetic time. More generally, ‘degree of difference’ scaling is a consequential choice for diversity estimation, testing and interpretation, and such scaling will warrant careful attention as we move forward.

Large NGS panels are now available [65–66], containing both synonymous (presumably neutral) and non-synonymous (possibly adaptive) substitutions [67–68]. Methods such as sequence capture of ultra-conserved elements (UCE’s) enable interspecific comparisons of evolutionary processes using standardized sequence datasets [69], and efforts are increasing to sort among myriad markers for smaller subsets that may represent important adaptive signals within and/or among the taxa examined [70–71]. With newer types of genetic markers becoming available, each with its own coding conventions, the choice of Euclidean metrics has obvious implications for diversity exposition.

### Translation between evolution and ecology

The use of multiple characters for quantitative taxonomic analysis dates to the 1960s [72], and has been a recurring theme in population genetics. More recently, there has been a suggestion to use taxonomic subdivision itself as a ‘degree of difference’ metric to quantify diversity, using simple code, say (*d*_*jk*_ = 0) for individuals in the same species, (*d*_*jk*_ = 1) for different species but same genus, and (*d*_*jk*_ = 2) for different genera [32]. Others have used more elaborate phylogenetic time depth estimates as ‘degree of difference’ metrics [45, 73–77]. There have also been increasing attempts to translate ecological separation into derivative evolutionary diversity outcomes [74–75, 77–89].

Both the need and our ability to communicate across the boundary between Evolution and Ecology continue to develop [90], and there should be three larger payoffs from what we have done here. (a) We have improved our ability to deal with ‘different is different’ coding, have scaled it [0,1], and have configured diversity analysis for convenient statistical evaluation. (b) By extending treatment of diversity into ‘degree of difference’ coding, we can attack problems where the scale of divergence itself is a part of the story. (c) Translation between diversity-metric and variance-metric methods provides access to a large panoply of quadratic estimation and testing methodology. Cross-disciplinary analytical translation will be of increasing importance and value, as evolutionary ecology continues to develop.

## Conclusion

We have here articulated a novel quadratic approach for partitioning genetic diversity within and among strata of a hierarchical sampling design that exhibits the desirable properties of diversity criteria. Importantly, this approach is unique among diversity treatments to date, in providing a statistical comparison of within-stratum diversity components at any given level. It also enables diversity analysis of a wide range of inter-individual genetic coding schemes that emerge from modern genomic work, as well as being extendable to organisms of virtually any ploidy level. This new approach promises to be informative and useful across a wide range of ecological and evolutionary studies.

### Statement on animal usage

The animal use protocols for *Antechinus* sampling and handling were covered by A2015/60 and A2012/49 permits (Australian National University).

### Accessibility arrangements

The *Antechinus* data are archived in Excel workbook form, along with listings of the QDiver results extracted from GenAlEx6.51 (http://biology.anu.edu.au/GenAlEx/). **D**^C^ data and analyses are presented in S5 Appendix, and **D**^R^ data and analyses are presented in S6 Appendix.

## Supporting information

S1 Appendix Partitioning within-population diversity into sub-components. (PDF)

S2 Appendix Scaling diversity components [0,1]. (PDF)

S3 Appendix Homogeneity testing of within-stratum diversity components. (PDF)

S4 Appendix Laboratory microsatellite protocols. (PDF)

S5 Appendix Analysis of Rao Diversity (QDiver) for D^C^ Matrix. (XLSX)

S6 Appendix Analysis of Rao Diversity (QDiver) for D^R^ Matrix. (XLSX)
