# Supplementary material for: Converting quadratic entropy to diversity: Both animals and alleles are diverse, but some are more diverse than others
Source: PLoS One. 2017 Oct 31;12(10):e0185499. doi: 10.1371/journal.pone.0185499 (PMC5663342; doi:10.1371/journal.pone.0185499)
Supplement: S1 Appendix — (PDF) [file pone.0185499.s001.pdf]

S1 Appendix: Partitioning within-population diversity into sub-components

With sexual diploids, there will be situations where uniting gametes will be either more (negative assortative) or less (positive assortative) diverse than expected under random mating. For any particular individual, say the jth, and scaling relative to (d^2_{max}), we compute a Q-value for the jth (2 x 2) within individual matrix

$$Q_{WI\text{-}j} = (\Sigma_{WI\text{-}j} / 2^2) \quad \rightarrow \quad \omega_{WI\text{-}j} = (1 - Q_{WI\text{-}j})^{-1} \quad . \qquad [S1.1]$$

The sampling within any single individual is too limited to provide useful replication for statistical testing of homogeneity, of course, but we can certainly obtain a weighted average (within-individual) diversity for any particular population, within which, the relative weight for each individual is $(4 / 4N_{Pk}) = (N_{Pk})^{-1}$, translating [S1.1] into population average form,

$$Q_{WI,Pk} = \text{average } (Q_{WI\text{-}j}) \text{ for population } P_k \quad \rightarrow \quad \omega_{WI,Pk} = (1 - Q_{WI,Pk})^{-1} . \qquad [S1.2]$$

To extract the inter-individual diversity within the kth population, we compute

$$\varepsilon_{AI,Pk} = (\alpha_{WPk} / \omega_{WI,Pk}) = [1 - Q_{WI,Pk}] / [\,1 - Q_{WPk}] = [1 - Q_{AI,Pk}]^{-1} \quad . \qquad [S1.3]$$

Just as the among-species (δ_{AS}) and among population (β_{AP}) diversity components can be back-translated into functions of the appropriate Q-values (Text Eqq. [7, 13]), we also have

$$Q_{AI,Pk} = [Q_{WPk} - Q_{WI,Pk}] / [1 - Q_{WI,Pk}] = (\varepsilon_{AI,Pk} - 1) / \varepsilon_{AI,Pk} \quad . \qquad [S1.4]$$

We can compute average among-individual (ε_{AI}) and within-individual (ω_{WI}) components, combining all the individuals within a single species, or even across the whole study, as needed. Finally, our partition of the total diversity in the study, takes the expanded form

$$\gamma = (\alpha_{WP} \cdot \beta_{AP} \cdot \delta_{AS}) = (\omega_{WI} \cdot \varepsilon_{AI} \cdot \beta_{AP} \cdot \delta_{AS}) \;. \qquad [S1.5]$$
