# Supplementary material for: Converting quadratic entropy to diversity: Both animals and alleles are diverse, but some are more diverse than others
Source: PLoS One. 2017 Oct 31;12(10):e0185499. doi: 10.1371/journal.pone.0185499 (PMC5663342; doi:10.1371/journal.pone.0185499)
Supplement: S2 Appendix — (PDF) [file pone.0185499.s002.pdf]

S2 Appendix: Scaling diversity components [0,1]

Jost [1] defined a [0,1]-scaled index of diversity overlap (labeled θ here) for any two elements of the study, which when applied (for example) to alleles within the total sample, yields [0,1] scaling, '0' for no sharing (total diversity) and '1' for total sharing (no diversity). Using (γ^*) here to denote the maximum possible value, we can write that scaling as:

$$0 \le \theta = [\ (\gamma)^{-1} - (\gamma^*)^{-1}\ ]\ /\ [\ 1 - (\gamma^*)^{-1}\ ] \le 1\ , \qquad [S2.1]$$

We have elsewhere [2] defined a complementary divergence index ($\gamma^{\sim}$), with '0' = (total sharing) and '1' = (non-overlap) for the total sample:

$$0 \le \gamma^{\sim} = (1 - \theta) = \cdots = \gamma^* \cdot (\gamma - 1)\ /\ (\gamma^* - 1) \cdot \gamma \le 1\ , \qquad [S2.2a]$$

which reduces the [0,1] - scaled diversity [S2.2a] to very convenient form:

$$[\ 0 \le \gamma^{\sim} = (Q\ /\ Q^*) \le 1\ ]\ . \qquad [S2.2b]$$

Similar [0,1]-scaling for each of nested diversity components ($\tilde{\delta}_{AS}$= among species, $\tilde{\sigma}_{WS}$ = within species, $\tilde{\beta}_{AP}$ = among populations, $\tilde{\alpha}_{WP}$ = within populations, $\tilde{\varepsilon}_{AI}$ = among individuals, and $\tilde{\omega}_{WI}$ = within individuals) yields analogous outcomes, specifically

Strata	Among-Strata	Within-Strata	
Species	$[\ 0 \le \tilde{\delta}_{AS} = (Q_{AS}\ /\ Q^*_{AS}) \le 1\ ]$	$[\ 0 \le \tilde{\sigma}_{WS} = (Q_{WS}\ /\ Q^*_{WS}) \le 1\ ]$,	[S2.3a]
Populations	$[\ 0 \le \tilde{\beta}_{AP} = (Q_{AP}\ /\ Q^*_{AP}) \le 1\ ]$	$[\ 0 \le \tilde{\alpha}_{WP} = (Q_{WP}\ /\ Q^*_{WP}) \le 1\ ]$,	[S2.3b]
Individuals	$[\ 0 \le \tilde{\varepsilon}_{AI} = (Q_{AI}\ /\ Q^*_{AI}) \le 1\ ]$	$[\ 0 \le \tilde{\omega}_{WI} = (Q_{WI}\ /\ Q^*_{WI}) \le 1\ ]$.	[S2.3c]

The Q^*-value maxima are strictly functions of the replication, determined by the sampling frame, and because that sampling frame does not change with permutational testing procedures, a test of the raw diversities (Text Eqq. [13] - [14], S1 Appendix Eq.[S1.3]) is monotonic and 1:1 with a test

of the correspoonding scaled ratios in Eqq. [S2.3a - S2.3c]. These scaled values, while not the raw diversities themselves, convey how large or small any estimated component may be, relative to how large or small it could be, given the sampling constraints. Conveniently, the entire panoply of diversity estimates (raw, maximum, and scaled) can be extracted directly from the raw **D**-matrix and array of sample sizes, via the corresponding Q and Q^* values.

$$\text{Estimate} = [1 - Q]^{-1} \qquad \text{Maximum} = [1 - Q^*]^{-1} \qquad \text{Scaled} = [\, Q / Q^* \,] \qquad \text{[S2.4]}$$

All of these diversity components are related to traditional heterozygosity-based variance analogs, and that fact leads to an array of non-parametric testing procedures of the within-stratum components (see S3 Appendix). Of more immediate interest here, the traditional heterozygosity-based variance ratios for the among-stratum components have been challenged as poorly bounded measures of among-stratum diversity. Using within-species (H_{WS}) and within-population (H_{WP}) heterozygosity to gauge divergence among-populations, Jost [3-4] defined an alternative [0,1]-scaled measure (D, here labeled Δ_{AP}) among K (equally weighted) populations

$$[\ 0 \leq \ \Delta_{AP} = K \cdot [\ H_{WS} - H_{WP}\ ] / (1 - H_{WP}) \cdot (K - 1) \leq 1\ ]\ , \qquad \text{[S2.5a]}$$

as a replacement for the traditional heterozygosity-based measures. We note that for ‘different is different’ ($\mathbf{D}^C$) coding, ($H_{WS} = Q_{WS}$), ($H_{WP} = Q_{WP}$), and for K equally replicated population samples (within a species), the maximum among-population value $Q^*_{AP} = (K - 1) / K$. For that same (equal replication) case, Eq. [S2.4a] translates (<u>exactly</u>) into

$$[\ 0 \leq \ \Delta_{AP} = \tilde{\beta}_{AP} = (Q_{AP} / Q^*_{AP}) \leq 1\ ]\ . \qquad \text{[S2.5b]}$$

The among-species analogue (G equally sampled species), translates into

$$[\ \Delta_{AS} = G \cdot [\ H - H_{WS}\ ] / (1 - H_{WS}) \cdot (G - 1) = \tilde{\delta}_{AS}\ ]\ , \qquad \text{[S2.6a]}$$

yielding

$$[\ 0 \leq \Delta_{AS} = \tilde{\delta}_{AS} = (Q_{AS} / Q^*_{AS}) \leq 1\ ]\ , \qquad \text{[S2.6b]}$$

and the among-individuals component (*N* diploid individuals), translates into

$$[\ \Delta_{AI} = N \cdot [\ H_{WP} - H_{WI}\ ] / (1 - H_{WI}) \cdot (N - 1) = \tilde{\varepsilon}_{AI}\ ]\ , \qquad [S2.7a]$$

yielding

$$[\ 0 \leq \Delta_{AI} = \tilde{\varepsilon}_{AI} = (Q_{AI} / Q^{*}_{AI}) \leq 1\ ]\ . \qquad [S2.7b]$$

For unbalanced sampling schemes, we use quadratic sample-size-weighted averages for both observed (Q) and maximal (Q^*) components, from top to bottom of the partition, but Eqq. [S2.5b, S2.6b, S2.7b] still apply. The advantages for bounded estimation of the among-population, among-species, and among-individual diversity components, relative to their more traditional variance component analogues, are the same as those shown for Jost's *D* [3-4].

We can extend all this to more general "degree of difference" case with $\mathbf{D}^{R}$ coding, in terms of the derivative Q and Q^*-values, yielding precisely the same outcomes, [S2.5b, S2.6b, S2.7b]. This treatment thus generalizes to the treatment to all among-stratum hierarchical layers, with or without balanced sampling, and with or without equi-distant alleles.

Literature Cited

1. Jost L, Partitioning diversity into independent alpha and beta components. Ecology. 2007;88: 2427–2439
2. Smouse PE, Whitehead MR, Peakall R, An informational diversity analysis framework, illustrated with sexually deceptive orchids in early stages of speciation. Molecular Ecol Resour**.** 2015;15: 1375-1384.
3. Jost L, *G*ST and its relatives do not measure differentiation. Molecular Ecol. 2008;17: 4015–4026.
4. Jost L, *D vs. G*ST: response to Heller and Siegismund (2009) and Ryman and Leimar (2009). Molecular Ecol. 2009;18: 2088–2091.
