# Supplementary material for: Converting quadratic entropy to diversity: Both animals and alleles are diverse, but some are more diverse than others
Source: PLoS One. 2017 Oct 31;12(10):e0185499. doi: 10.1371/journal.pone.0185499 (PMC5663342; doi:10.1371/journal.pone.0185499)
Supplement: S3 Appendix — (PDF) [file pone.0185499.s003.pdf]

S3 Appendix: Homogeneity testing of within-stratum diversity components

If we scale our distance matrix **D** by its maximum entry (d_{max}^2), then average the elements, both for the total collection and for any relevant subdivisions (e.g., within single species, within single populations, or even within single individuals), we can translate each of the resulting Q-values into a corresponding sum of squares. For the grand total collection of ($2N$) alleles in the study, for the ($2N_{Sg}$) alleles within the gth species (g = 1, ... , G), or for the ($2N_{Pk}$) alleles within the kth population (k = 1, ... , K), the corresponding Q - values are simple multiples of the corresponding sums of squares and variances:

$$Q = \left({}_{sum}\mathbf{D}/4N^2\right) = (\mathrm{SS} / N) = \mathrm{V} \cdot (2N - 1) / N \quad , \qquad \text{[S3.1a]}$$

where SS and V are the grand total sum of squares and total variance, respectively. Similar extractions for the within-species, within-population, and within-individual Q-values yield

$$Q_{\mathrm{WS}g} = \cdots = (\mathrm{SS}_{\mathrm{WS}g} / N_{\mathrm{S}g}) = \mathrm{V}_{\mathrm{WSg}} \cdot (2N_{\mathrm{S}g} - 1) / N_{\mathrm{S}g} \quad , \qquad \text{[S3.1b]}$$

$$Q_{\mathrm{WP}k} = \cdots = (\mathrm{SS}_{\mathrm{WP}k} / N_{\mathrm{P}k}) = \mathrm{V}_{\mathrm{WP}k} \cdot (2N_{\mathrm{P}k} - 1) / N_{\mathrm{P}k} \quad , \qquad \text{[S3.1c]}$$

$$Q_{\mathrm{WI}j} = \cdots = (\mathrm{SS}_{\mathrm{WI}\text{-}j} / N_{\mathrm{I}j}) = \mathrm{V}_{\mathrm{WI}j} \cdot (2N_{\mathrm{I}j} - 1) / N_{\mathrm{I}j} \quad . \qquad \text{[S3.1d]}$$

Averages of the within-stratum components of [S3.1b - S3.1d] can be computed as needed.

We convert Q-values to their diversity analogues, and then use their maxima to scale each of those components from (0 = no diversity) to (1 = maximum diversity possible). Given the sampling realities of the study, those maxima take very simple forms,

$$Q^* = (2N - 1) / 2N \qquad Q^*_{\mathrm{WS}g} = (2N_{\mathrm{S}g} - 1) / 2N_{\mathrm{S}g} \quad ,$$

$$Q^*_{\mathrm{WP}k} = (2N_{\mathrm{P}k} - 1) / 2N_{\mathrm{P}k} \qquad Q^*_{\mathrm{WI}-j} = (2N_{\mathrm{I}j} - 1) / 2N_{\mathrm{I}j} = (\tfrac{1}{2}) \quad , \qquad \text{[S3.2]}$$

recalling (for diploids) that $\left(N_{\mathrm{I}j} = 2\right)$ alleles.

The net result of such translation is we translate all of our within-stratum diversity components into analogous within-stratum variance analogues,

$$\gamma^{\sim} = [\, Q \,/\, Q^{*} \,] = 2 \cdot \mathrm{V} \qquad \sigma^{\sim}_{\mathrm{WSg}} = [\, Q_{\mathrm{WS}g} \,/\, Q^{*}_{\mathrm{WS}g}] = 2 \cdot \mathrm{V}_{\mathrm{WS}g} \,,$$

[S3.3]

$$\alpha^{\sim}_{\mathrm{WP}k} = [\, Q_{\mathrm{WP}k} \,/\, Q^{*}_{\mathrm{WP}k} \,] = 2 \cdot \mathrm{V}_{\mathrm{WP}k} \qquad \omega^{\sim}_{\mathrm{WI}-j} = [\, Q_{\mathrm{WI}-j} \,/\, Q^{*}_{\mathrm{WI}-j} \,] = 2 \cdot \mathrm{V}_{\mathrm{WI}\text{-}j} \,.$$

Given a collection of G species, we will typically compute a weighted average value for (pooled) within-species $\left(\sigma^{\sim}_{\mathrm{WS}g}\right)$ diversity, where the sampling weights (f_{Sg}-values) for the G species are defined as in Text Eq. [5]. Straightforward algebraic manipulation yields

$$\sigma^{\sim}_{\mathrm{WS}} = (f_{\mathrm{SA}} \cdot Q_{\mathrm{SA}} + \cdots + f_{\mathrm{SG}} \cdot Q_{\mathrm{SG}}) \,/\, (f_{\mathrm{SA}} \cdot Q^{*}_{\mathrm{SA}} + \cdots + f_{\mathrm{SG}} \cdot Q^{*}_{\mathrm{SG}}) \;=\; \cdots$$

[S3.4a]

$$= 2 \cdot (\eta_{\mathrm{SA}} \cdot \mathrm{V}_{\mathrm{SA}} + \cdots + \eta_{\mathrm{SG}} \cdot \mathrm{V}_{\mathrm{SG}}) = 2 \cdot \mathrm{V}_{\mathrm{WS}} \,,$$

where the sample-frame-dependent (η) - weights take the form

$$\eta_{\mathrm{S}g} = N_{\mathrm{S}g} \cdot (2N_{\mathrm{S}g} - 1) \,/\, [\, N_{\mathrm{SA}} \cdot (2\, N_{\mathrm{SA}} - 1) + \cdots + N_{\mathrm{SG}} \cdot (2N_{\mathrm{SG}} - 1) \,] \,, \qquad \text{[S3.4b]}$$

with the η-weights summing to unity. Thus, ($\sigma^{\sim}_{\mathrm{WS}} = 2{\cdot}\mathrm{V}_{\mathrm{WS}}$) is a weighted-average within-species scaled diversity component. Averaging of K within-population diversity components, using the sort of $f_{\mathrm{P}k}$-weights in Text Eq. [10], yields (weighted average) within-population translation,

$$\alpha^{\sim}_{\mathrm{WP}} = (f_{\mathrm{P1}} \cdot Q_{\mathrm{WP1}} + \cdots + f_{\mathrm{PK}} \cdot Q_{\mathrm{WPK}}) \,/\, (f_{\mathrm{P1}} \cdot Q^{*}_{\mathrm{WP1}} + \cdots + f_{\mathrm{PK}} \cdot Q^{*}_{\mathrm{WPK}}) = \cdots$$

[S3.5a]

$$= 2 \cdot (\eta_{\mathrm{P1}} \cdot \mathrm{V}_{\mathrm{WP1}} + \cdots + \eta_{\mathrm{PK}} \cdot \mathrm{V}_{\mathrm{WPK}}) = 2 \cdot \mathrm{V}_{\mathrm{WP}} \,,$$

where the sample-frame imposed population weights (η-values) now take the forms

$$\eta_{\mathrm{P}k} = N_{\mathrm{P}k} \cdot (2N_{\mathrm{P}k} - 1) \,/\, [\, N_{\mathrm{P1}} \cdot (2\, N_{\mathrm{P1}} - 1) + \cdots + N_{\mathrm{PK}} \cdot (2N_{\mathrm{PK}} - 1) \,] \,, \qquad \text{[S3.5b]}$$

with those (η-weights) again summing to unity, and yielding ($\alpha^{\sim}_{\mathrm{WP}} = 2{\cdot}\mathrm{V}_{\mathrm{WP}}$) as the weighted-average within-population scaled diversity.

Multi-stratum averages are convenient summaries, but there are situations where those averages conceal more than they reveal. We can test for heterogeneity of within-stratum diversity components with a non-parametric analogue of Bartlett's test of homogeneous within-stratum variances [1]. A test of homogeneous within-species diversity is tantamount to a test of homogeneous within-species variances, with a null hypothesis of the form:

$$(\sigma_{\widetilde{\mathrm{WSA}}} = \cdots = \sigma_{\widetilde{\mathrm{WSG}}}) \quad \Leftrightarrow \quad (V_{\mathrm{WSA}} = \cdots = V_{\mathrm{WSG}}) \ . \qquad \text{[S3.6a]}$$

The traditional Bartlett's test takes the form:

$$\text{Bartlett's Test} = [\ (2N_{\mathrm{SA}} - 1) \cdot \ln (V_{\mathrm{WSA}}) + \cdots + (2N_{\mathrm{SG}} - 1) \cdot \ln (V_{\mathrm{WSG}})\ ] - (2N - \mathrm{G}) \cdot \ln (V_{\mathrm{WS}}) \ , \qquad \text{[S3.6b]}$$

which is typically compared with an asymptotic chi-square criterion [2]. The QDIVER routine (instead) permutes variance contributions of individual alleles (each measured from its own species mean) freely among species, computing a value of [S3.6b] for each randomization, and comparing the realized data outcome against the distribution of (say 999) permutational trials.

To deploy [S3.6b], we start by extracting the diversity contribution of each allele to the within-species (diversity, in variance form). Start with the $\mathbf{D}_{\mathrm{WS}g}$-matrix, of dimension ($2N_{\mathrm{S}g}$ x $2N_{\mathrm{S}g}$), and scale it both by the largest element (d^2_{max}) and the maximum $(Q^*_{\mathrm{WS}g})$-value, yielding

$$\mathbf{D}_{\widetilde{\mathrm{S}g}} = 2N_{\mathrm{S}g} \cdot \mathbf{D}_{\mathrm{S}g} \,/\, (2\, N_{\mathrm{S}g} - 1) \cdot (d^2_{max}) \ , \qquad \text{[S3.7a]}$$

designed to scale [$0 \leq \sigma_{\widetilde{\mathrm{S}g}} \leq 1$] . We can extract the squared individual deviation of each allele for the (g^{th})-species mean, by using a Gower transformation [3] of the distance matrix in [S3.7a] to its corresponding covariance matrix form. Conveniently, we only need the diagonal (variance) elements of that covariance matrix ($\mathbf{C}_g$), so for the jth allele, we compute:

v_{jj} = (½) · [j^{th} row aver. of $\mathbf{D}_{\tilde{S}g}$ + j^{th} column aver. of $\mathbf{D}_{\tilde{S}g}$ – global aver. of $\mathbf{D}_{\tilde{S}g}$] , [S3.7b]

and the summation of (v_{jj}), over all ($2N_{Sg}$) alleles within the gth species, is the variance within that species (V_{WSg}). We perform a separate transform for each of the G species under consideration, and from each of them extract the ($2N_{Sg}$) single-allele contributions to the within-species variation. We permute all $2N$ allelic (v_{jj}) values among species, in the numbers actually sampled, recompute the V_{WSg} and the (pooled) V_{WS} values for each of 999 trials, deploy the data as the 1,000th randomization, and evaluate Eq. [S3.6b] for each trial.

We can test for homogeneous within-population diversities in exactly the same fashion. The null hypothesis is that the within-population diversities/variances are homogeneous

$$(\alpha_{\tilde{W}P1} = \cdots = \alpha_{\tilde{W}PK}) \quad \Leftrightarrow \quad (V_{WP1} = \cdots = V_{WPK}) \ . \qquad [S3.8a]$$

To test this hypothesis, we again compute a Bartlett's test, now defined as

$$\text{Bartlett's Test} = [\ (2N_{P1} - 1) \cdot \ln (V_{WP1}) + \cdots + (2N_{PK} - 1) \cdot \ln (V_{WPK})\ ] - (2N - K) \cdot \ln (V_{WP}) \ , \qquad [S3.8b]$$

where deviations of individual alleles are now measured from their population means. Again converting a distance matrix to its covariance matrix analogue for each population separately, and scaling, we obtain

$$\mathbf{D}_{\tilde{P}k} = 2N_{Pk} \cdot \mathbf{D}_{Pk} / (2\ N_{Pk} - 1) \cdot (d^2_{max}) \ . \qquad [S3.9a]$$

Thus, we scale [$0 \leq \alpha_{\tilde{P}k} \leq 1$], and then extract the squared individual deviation of each allele from its own population mean as

v_{jj} = (½) · [j^{th} row aver. of $\mathbf{D}_{\tilde{P}k}$ + j^{th} column aver. of $\mathbf{D}_{\tilde{P}k}$ – global aver. of $\mathbf{D}_{\tilde{P}k}$] , [S3.9b]

and permute those v_{jj}-values freely among populations, computing individual within-population

variances, as well as the pooled within-population variance, using the observed values as the 1,000th randomization, and evaluating the null distribution of [S3.8b] for significance.

We treat scaled within-individual diversity in the same fashion, for situations where it would be useful to query whether within-individual diversity is homogeneous (or not) among individuals, by permuting v_{jj}-values among individuals. The larger point is that Homo- vs Heterogeneous (within-stratum diversity) is tantamount to Homo- vs Heterogeneous (within-stratum variance), so we have a convenient statistical comparison of homogeneous vs heterogeneous diversity hypotheses within different strata at any given level of the hierarchy.

References

1. Bartlett MS, Properties of sufficiency and statistical tests. Proc Roy Stat Soc. 1937;160: 268-282.
2. Snedecor GW, Cochran WG, Statistical Methods, Eighth Edition, Iowa State Univ Press, Ames, Iowa (USA). 1989.
3. Gower JC, Some distance properties of latent root and vector methods used in multivariate analysis. Biometrika. 1966;53: 315-328.
