# Supplementary material for: Converting quadratic entropy to diversity: Both animals and alleles are diverse, but some are more diverse than others
Source: PLoS One. 2017 Oct 31;12(10):e0185499. doi: 10.1371/journal.pone.0185499 (PMC5663342; doi:10.1371/journal.pone.0185499)
Supplement: S4 Appendix — (PDF) [file pone.0185499.s004.pdf]

S4 Appendix: Laboratory microsatellite protocols

DNA extraction and genotyping of *Antechinus* samples

We extracted DNA from all samples using Qiagen 96-well plate DNeasy extraction kits. We genotyped all antechinus samples with the microsatellite loci Aa2B, Aa2E, Aa2G, Aa4A, Aa4K, Aa7D, Aa7F and Aa7M [1-2], as well as a Y chromosome SRY locus [3], not used for this paper. PCR amplification conditions are presented in Table S4.1 and protocols followed those in the papers describing the primer sequences themselves [1-2], except that each forward primer was tagged with the (-21) M13 sequence (TGTAAAACGACGGCCAGT) to enable dye-labelling of PCR products, using the approach of Schuelke [4]. Following amplification, PCR products were run on an ABI3100 sequencer and scored with GeneMapper software at AGRF laboratories. The microsatellite panels used here were the best-performing subset of loci from those previously used for these species in these regions [1, 5-6].

Table S4.1. PCR amplification conditions for the eight microsatellite loci used in this study, originally described by [1-2].

Locus	Primer	DNA Sequence	Annealing Temperature	[$MgCl_2$] (mM)
Aa2B	Forward	GTACCACAAGATGCACCTAC	54	1.5
	Reverse	TTCACAGCCTAACTAATGCTCCTA		
Aa2E	Forward	TCTCGGCTCCTGTCAGTT	54	2
	Reverse	TCACATAGGGCAGCTTTCCTCCTG		
Aa2G	Forward	TTACACACATGCCCATTCAC	50	2
	Reverse	AGTTCTAAAACAGAGGTTCTT		
Aa4A	Forward	TTTGATCCTCAGAGACTTGAT	50	2.25
	Reverse	CCAAATCTAAAATATCC		

Table S4.1. (continued)

Locus	Primer	DNA Sequence	Annealing Temperature	[$MgCl_2$] (mM)
Aa4K	Forward	TCTGTGGAGCCTCTAGAGAAT	50	2
	Reverse	AAGAGGATAACCCATTCAGA		
Aa7D	Forward	GGATTTGATCTCAGGTTTTC	54	2.5
	Reverse	ATATCCACCAATGACTGCAA		
Aa7F	Forward	ATTGCCTGTTGCTACCAT	50	2.5
	Reverse	ACACATAACTCCTAAGAATTCCT		
Aa7M	Forward	TGCTTTGTTCTTGCTAAGTA	50	2
	Reverse	ACAATCATATGTTTATGTAGCC		

References

1. Banks SC, Finlayson GR, Lawson SJ, Lindenmayer DB, Paetkau D, Ward SJ, Taylor AC. The effects of habitat fragmentation due to forestry plantation establishment on the demography and genetic variation of a marsupial carnivore, *Antechinus agilis*. Biol. Conservation. 2005;122: 581-597.
2. Kraaijeveld-Smit FJL, Ward SJ, Temple-Smith PD, Paetkau D. Factors influencing paternity success in *Antechinus agilis*: last-male sperm precedence, timing of mating and genetic compatibility. J. Evolutionary Biol. 2002;15: 100-107.
3. Watson CM, Margan SH, Johnston PG. Sex-chromosome elimination in the bandicoot *Isoodon macrourus* using Y-linked markers. Cytogenet. Cell Genetics. 1998;81: 54-59.
4. Schuelke M. An economic method for the fluorescent labeling of PCR fragments. Nature Biotechology. 2000;18: 233-234.
5. Banks SC, McBurney L, Blair D, Davies ID, Lindenmayer DB. Where do animals come from during post-fire population recovery? Implications for ecological and genetic patterns in post-fire landscapes. Ecography. 2017; DOI: 10.1111/ecog.02251.
6. Kraaijeveld-Smit FJL, Lindenmayer DB, Taylor AC, MacGregor C, Wertheim B. Comparative genetic structure reflects underlying life histories of three sympatric small mammal species in continuous forest of south-eastern Australia. Oikos. 2007;116: 1819-1830.
